# Supplementary material for: Defective STIM-mediated store operated Ca2+ entry in hepatocytes leads to metabolic dysfunction in obesity
Source: eLife. 2017 Dec 15;6:e29968. doi: 10.7554/eLife.29968 (PMC5777820; doi:10.7554/eLife.29968)

Figure 1B

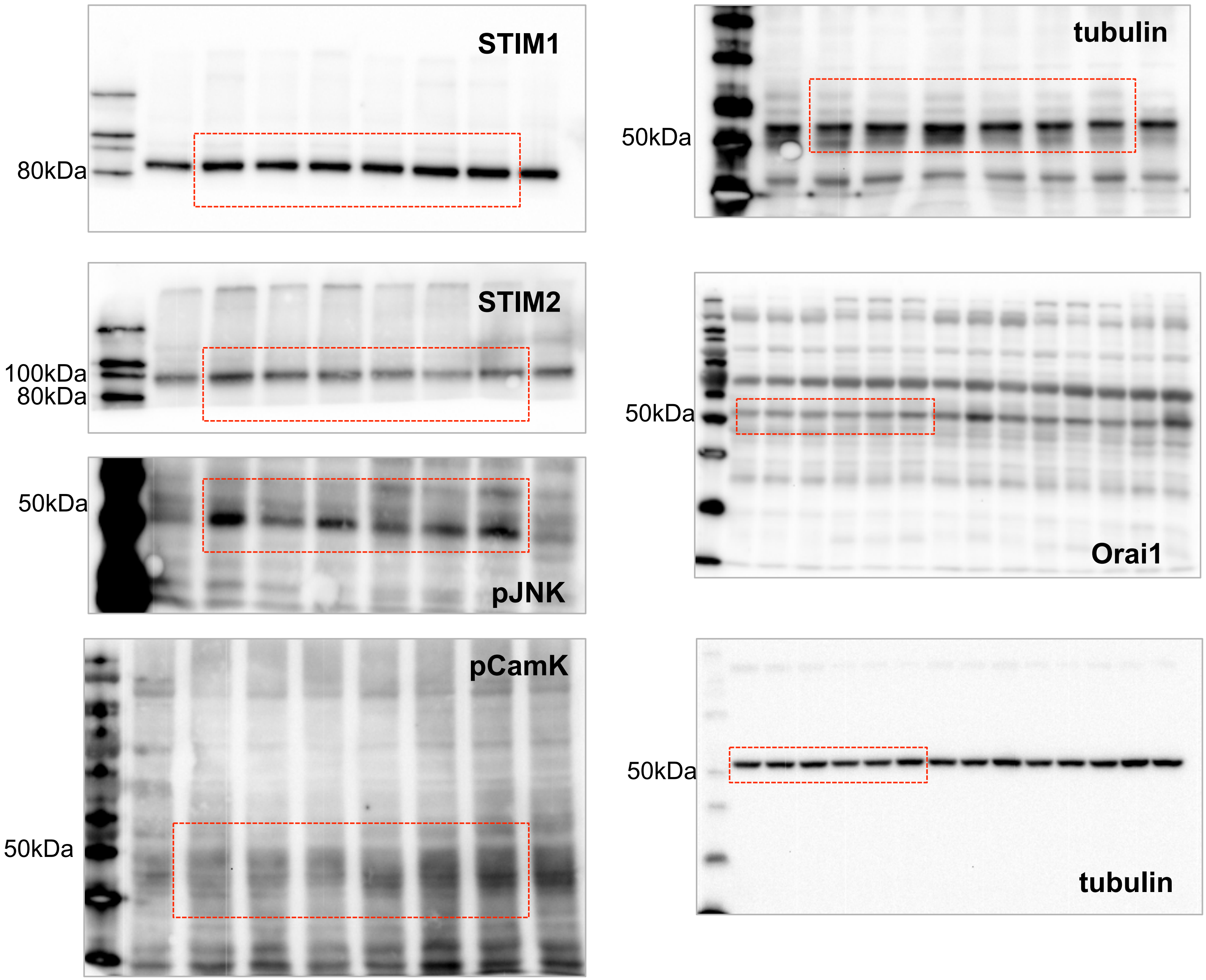
STIM1
80kDa
tubulin
50kDa
STIM2
100kDa
80kDa
50kDa
Orai1
50kDa
pJNK
pCamK
50kDa
50kDa
tubulin

Figure 1S1B

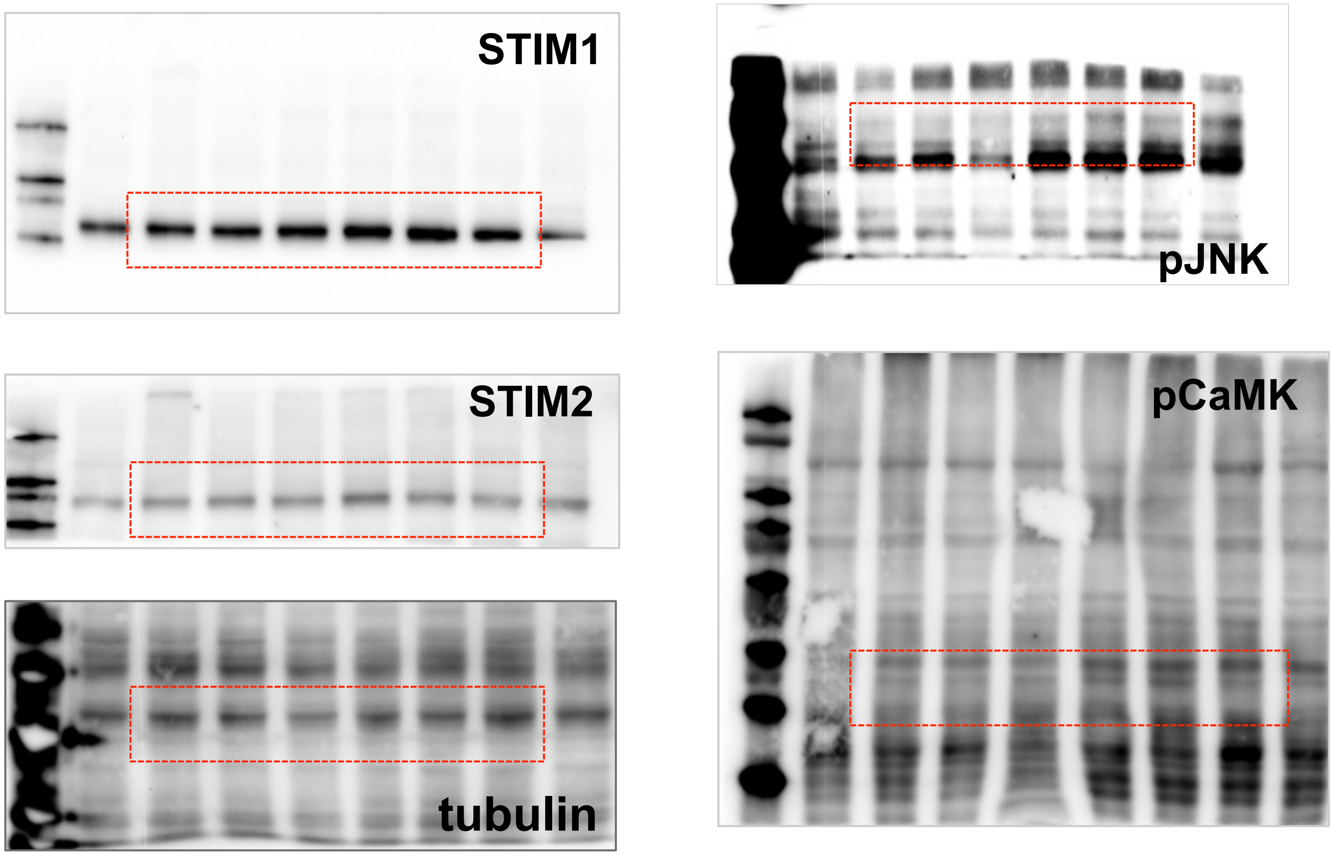

Figure 2A

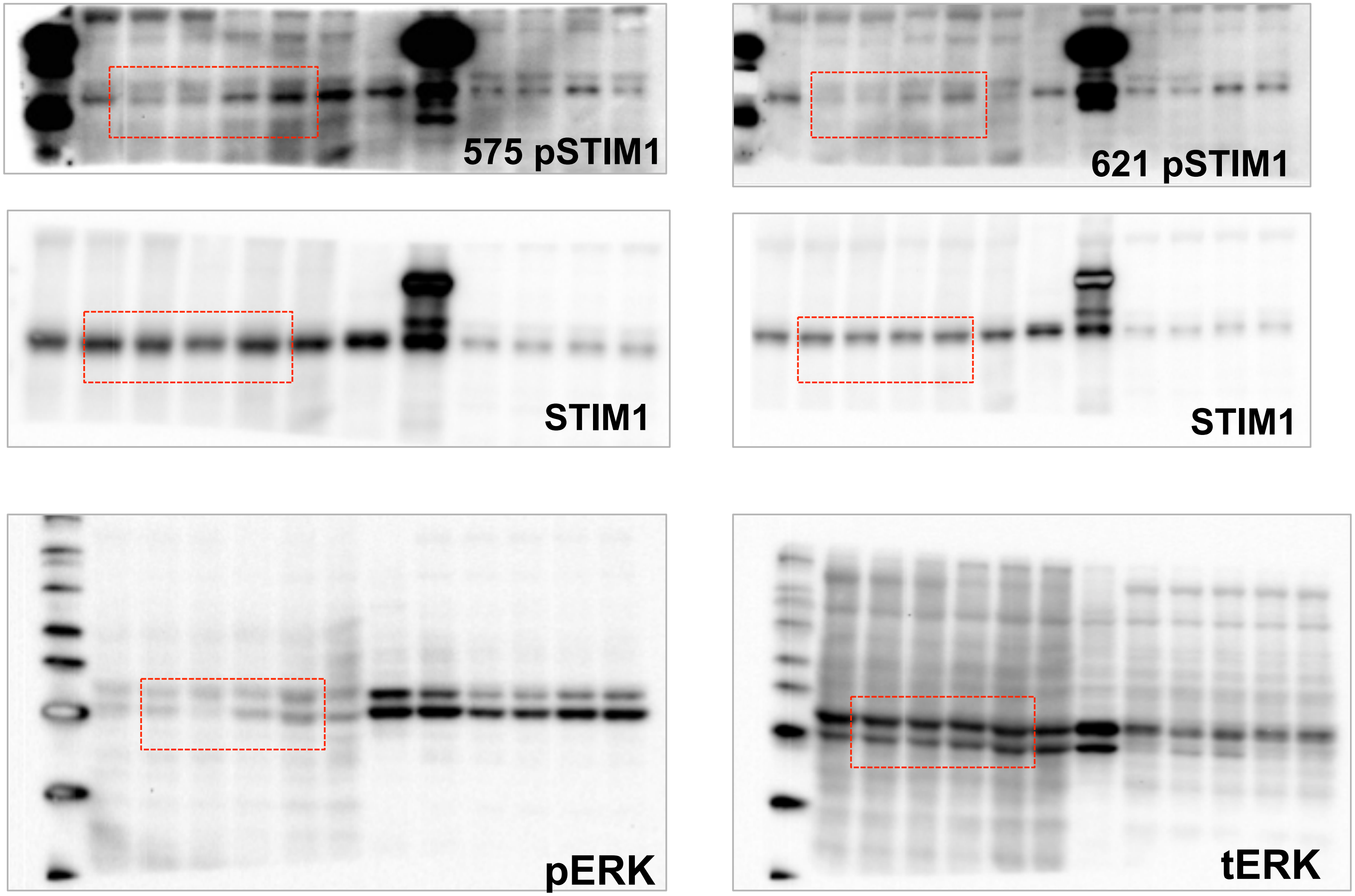

Figure 2B

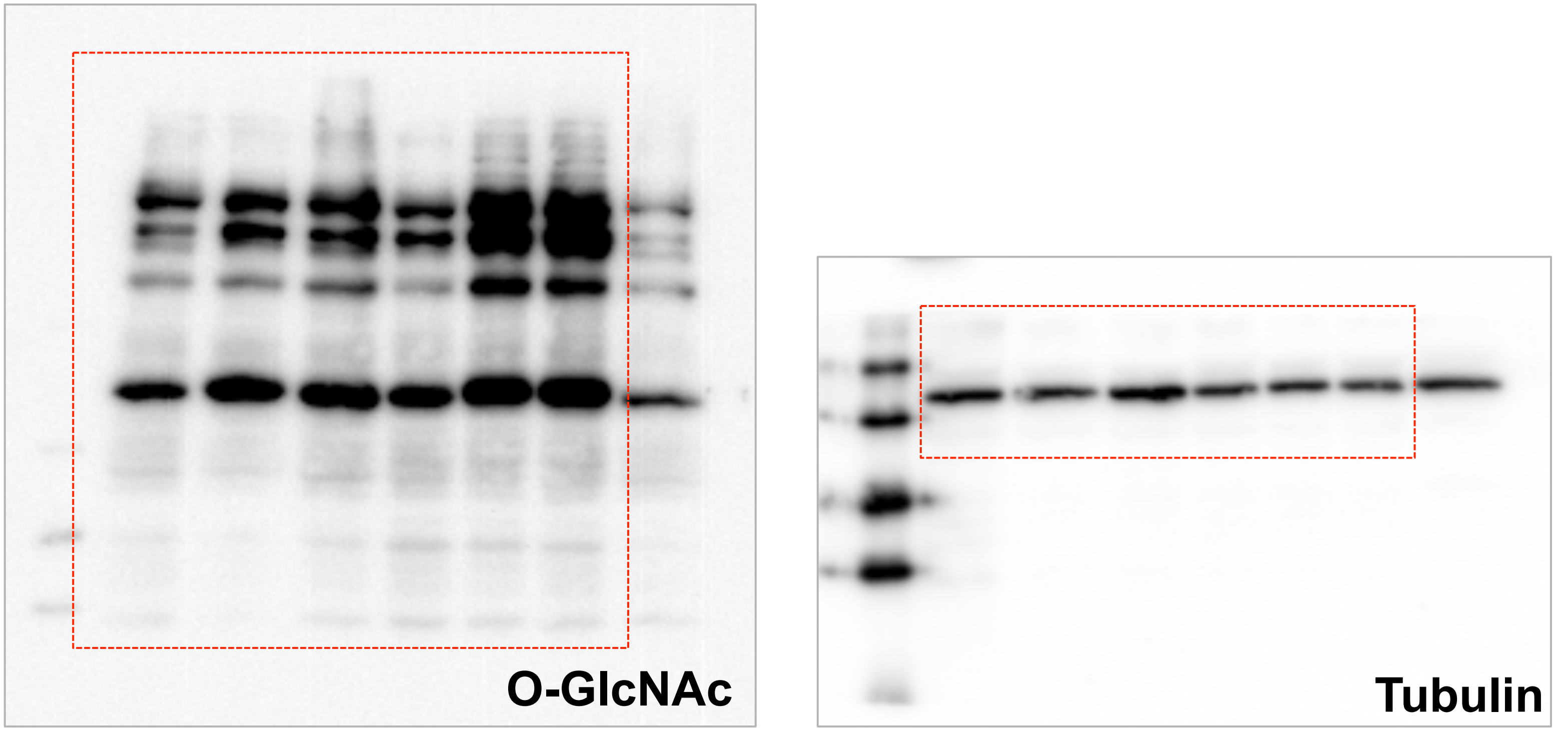

Figure 2C

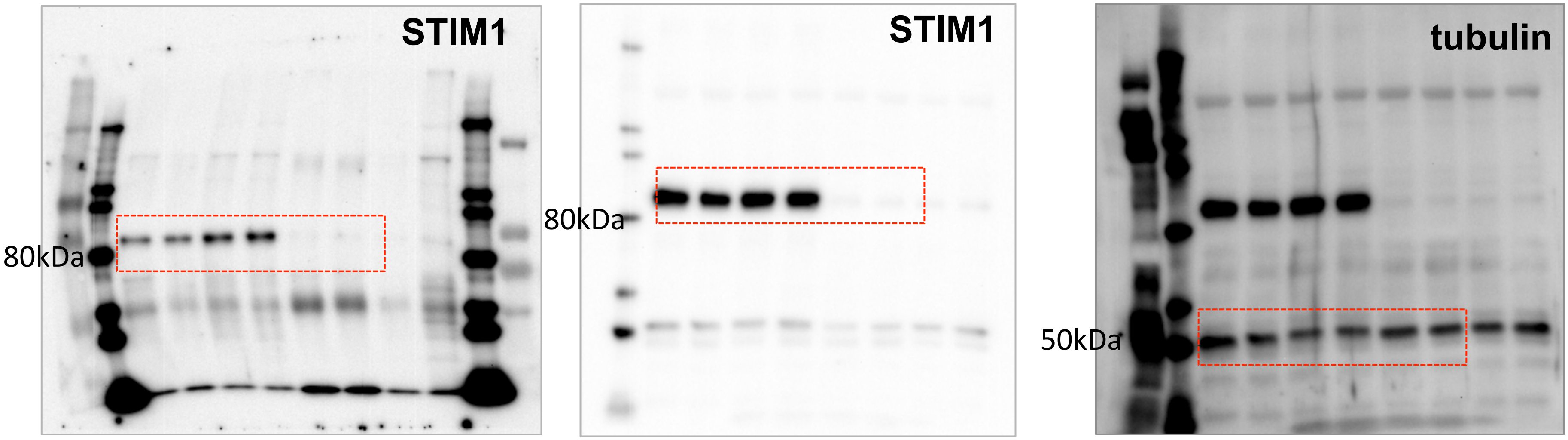

Figure 2D

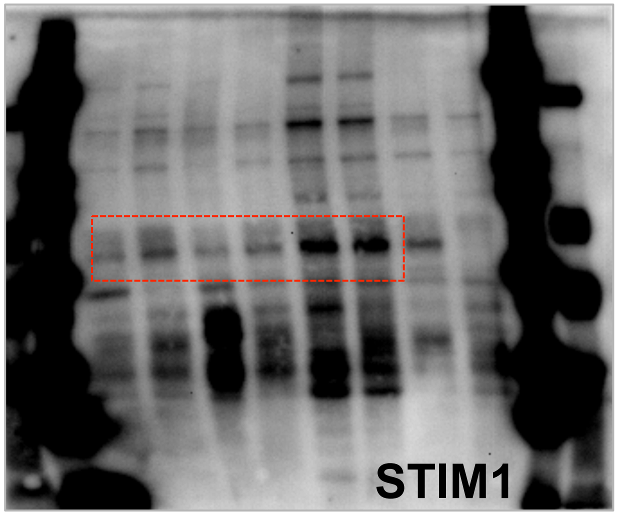

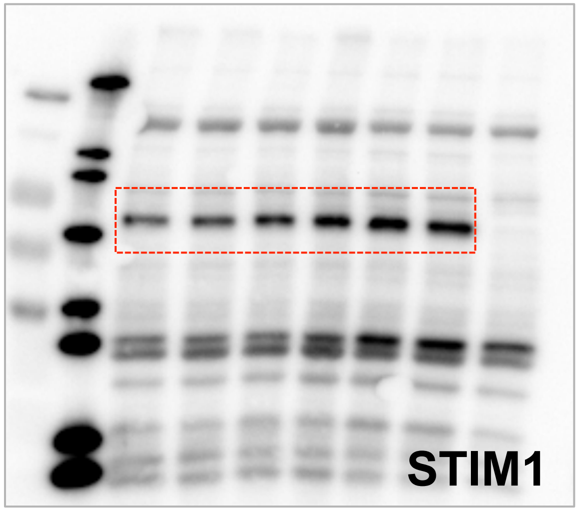

Silver stain

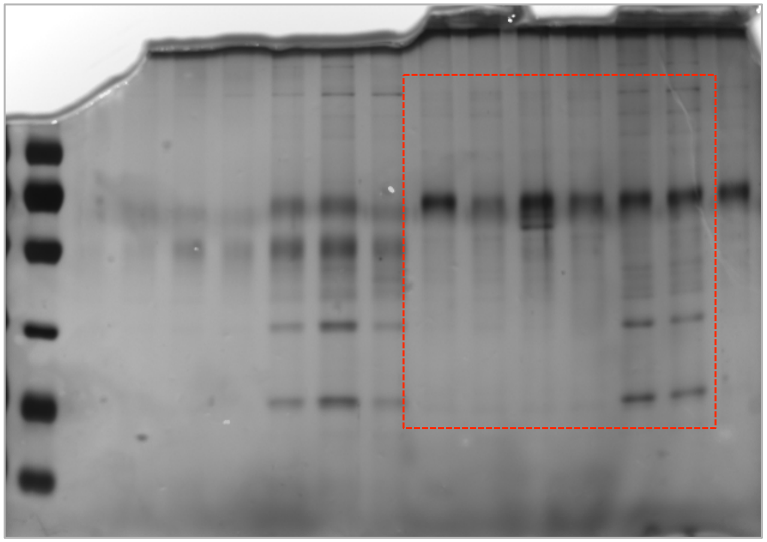

Figure 2E

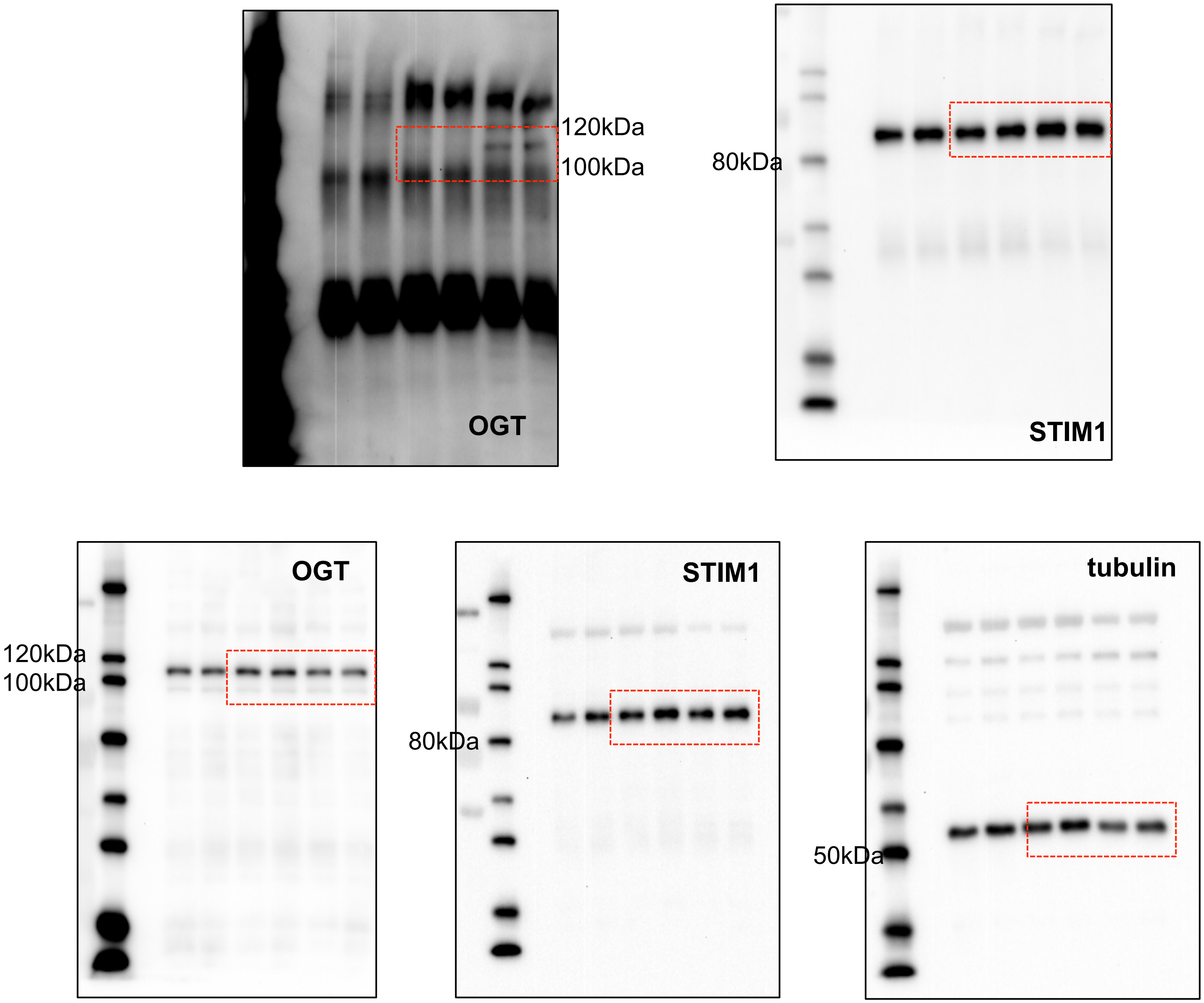

120kDa
100kDa
OGT
80kDa
STIM1
120kDa
100kDa
OGT
80kDa
STIM1
50kDa
tubulin

Figure 2F

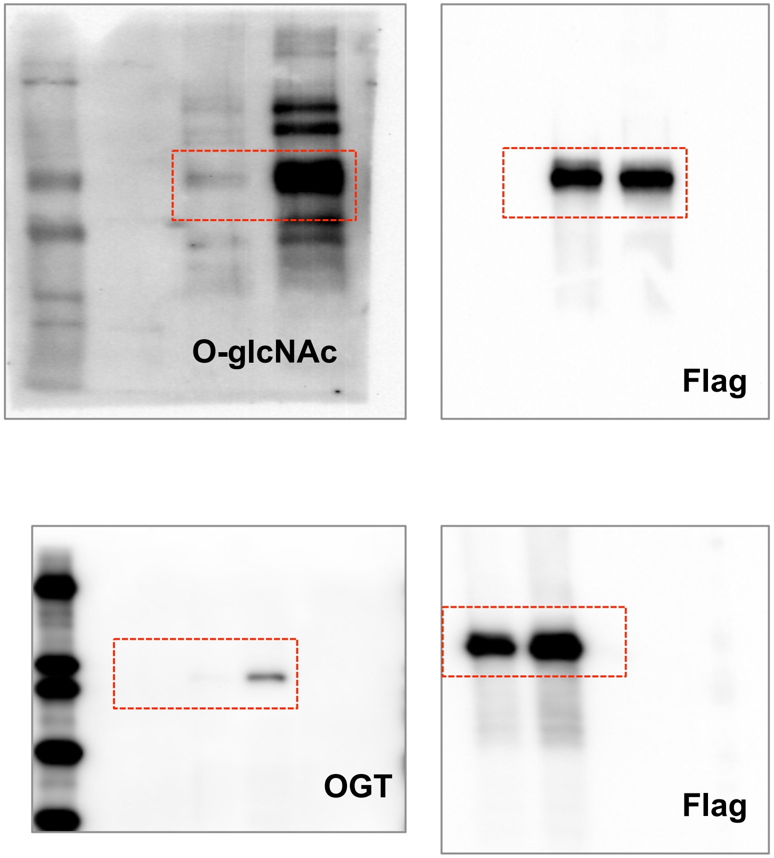

Figure 2K

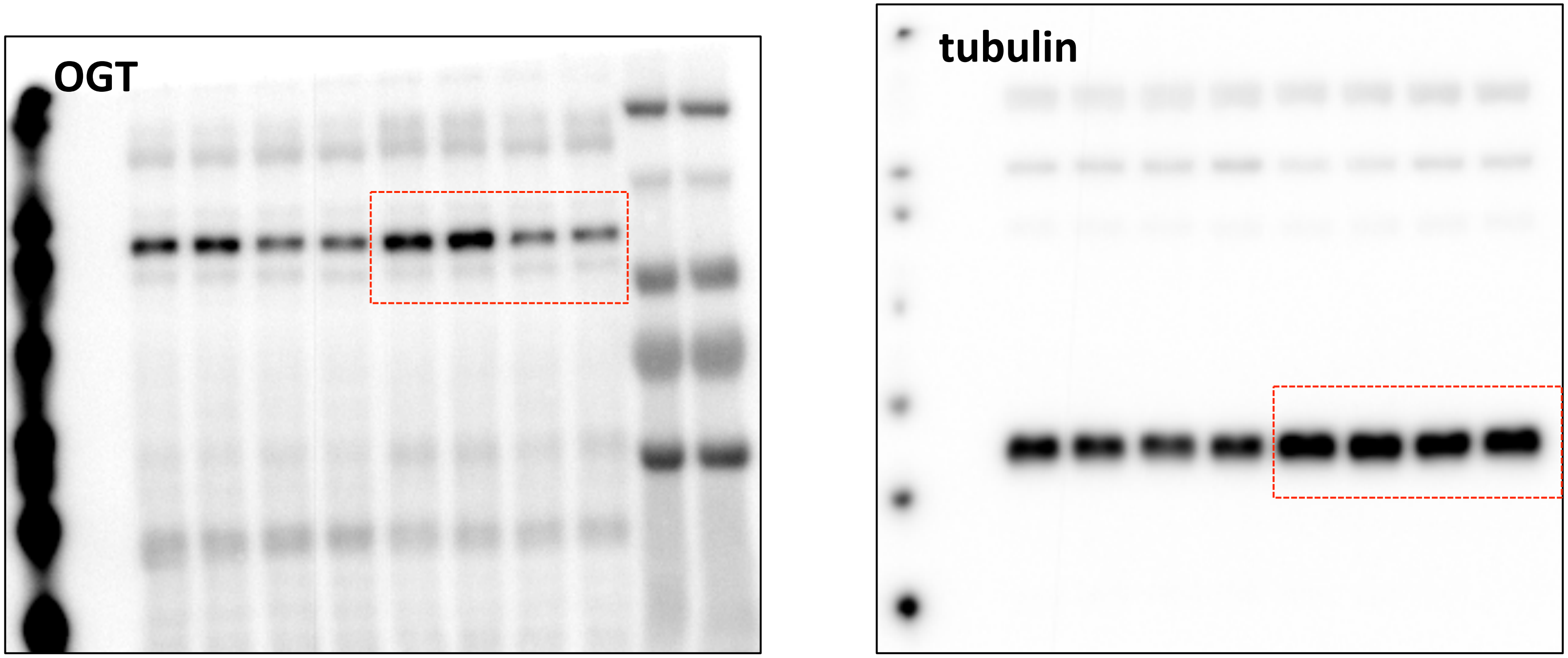

Figure 2L

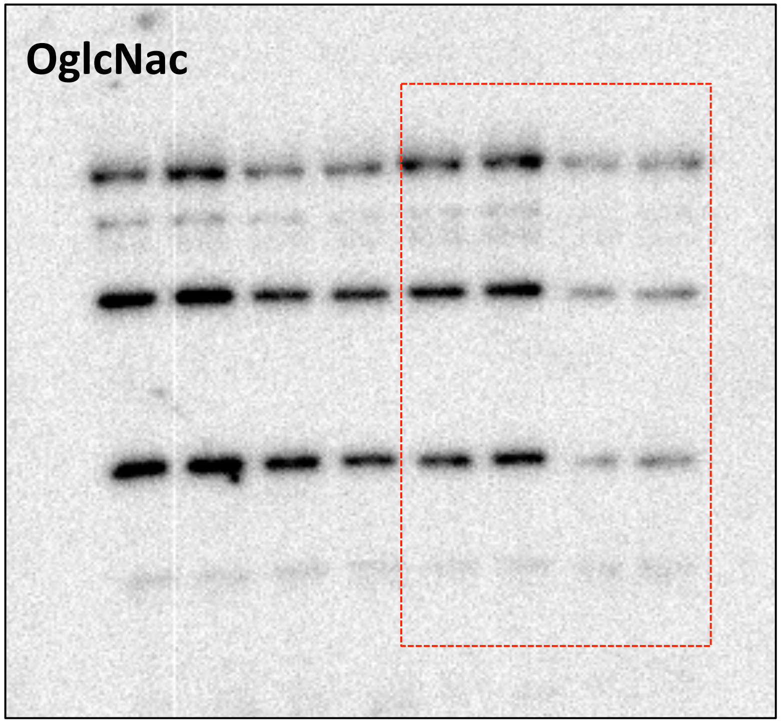

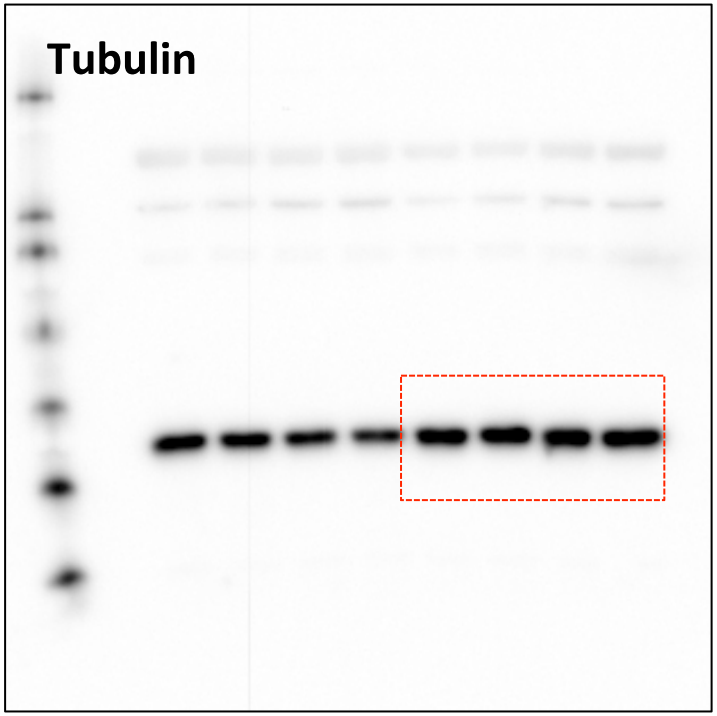

Figure S2A

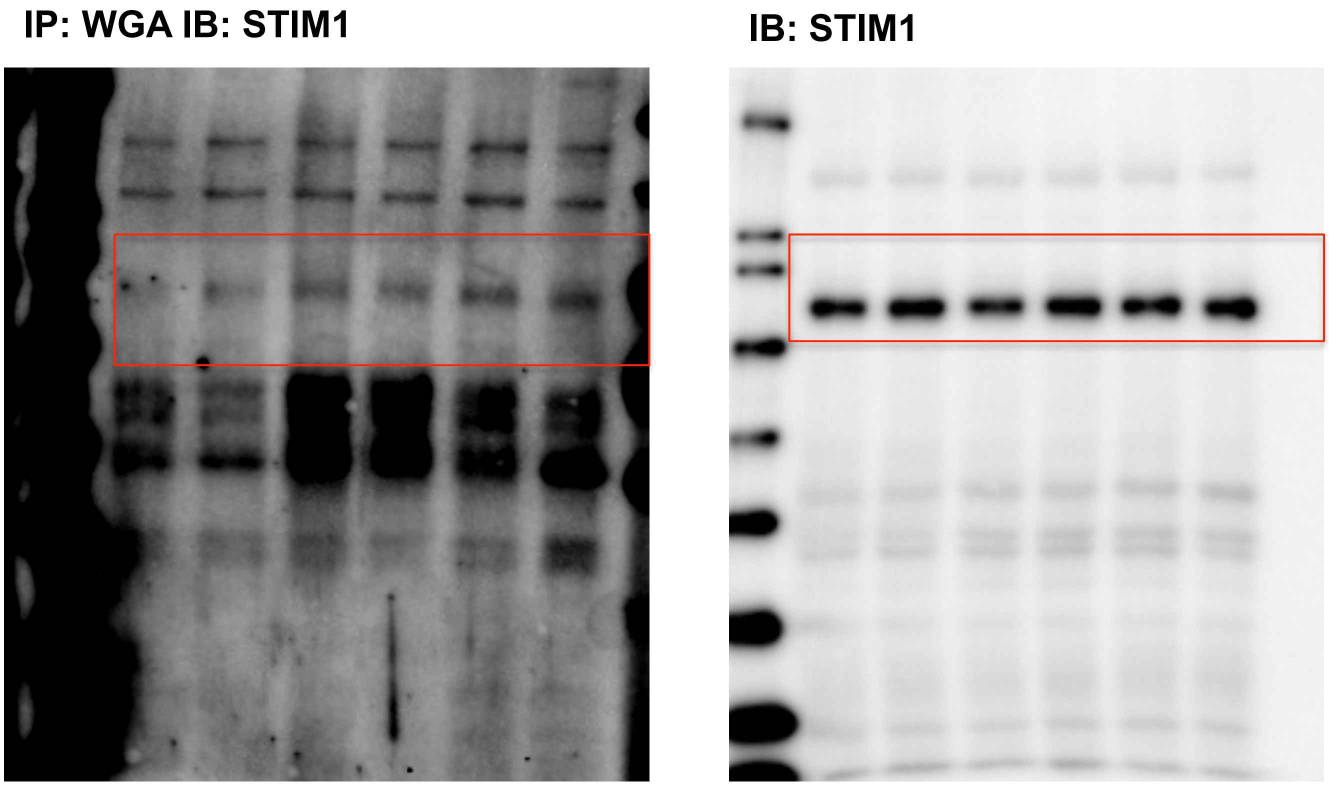

Figure S2B

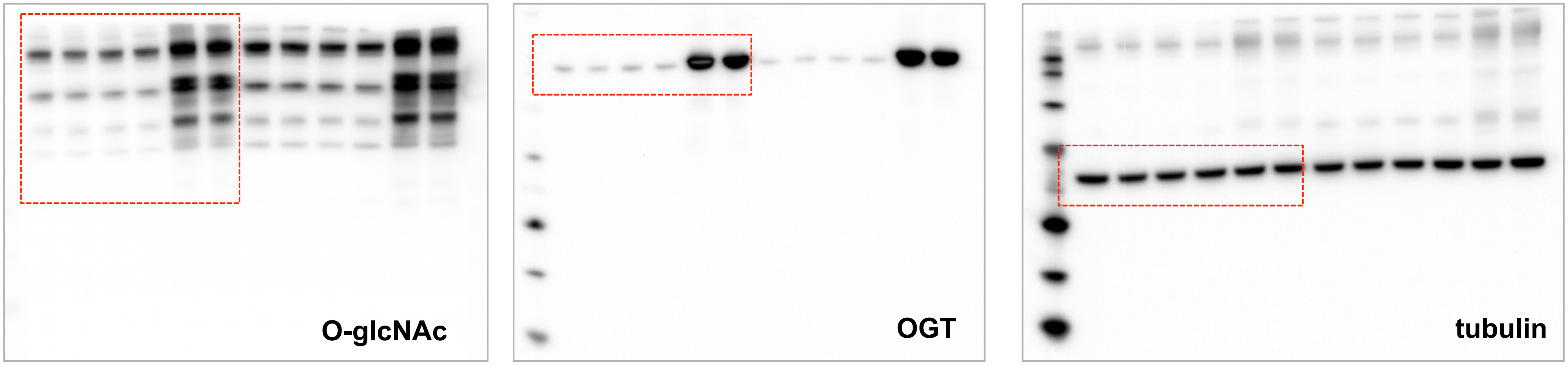

Figure S2C

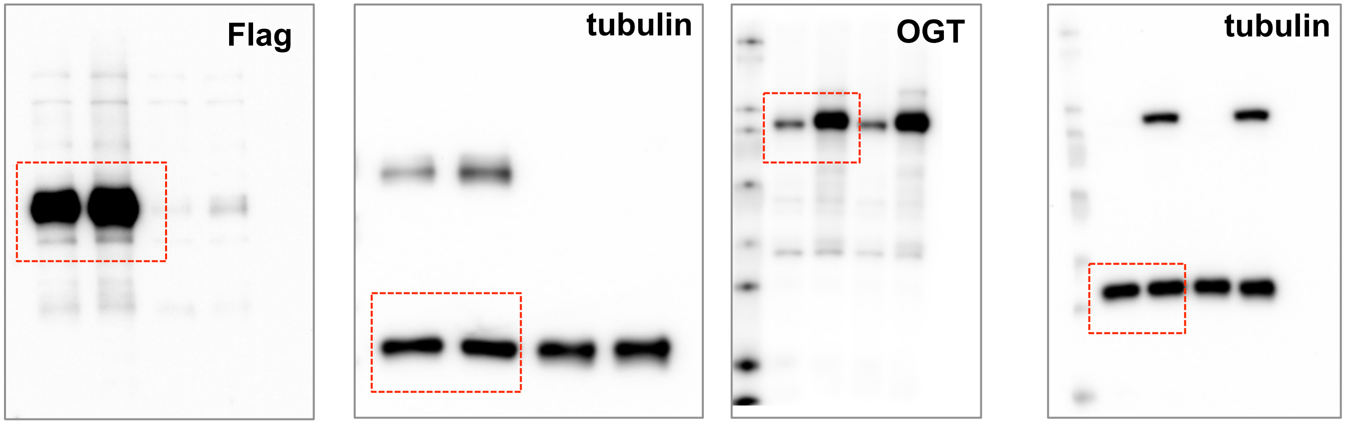

Figure 3B

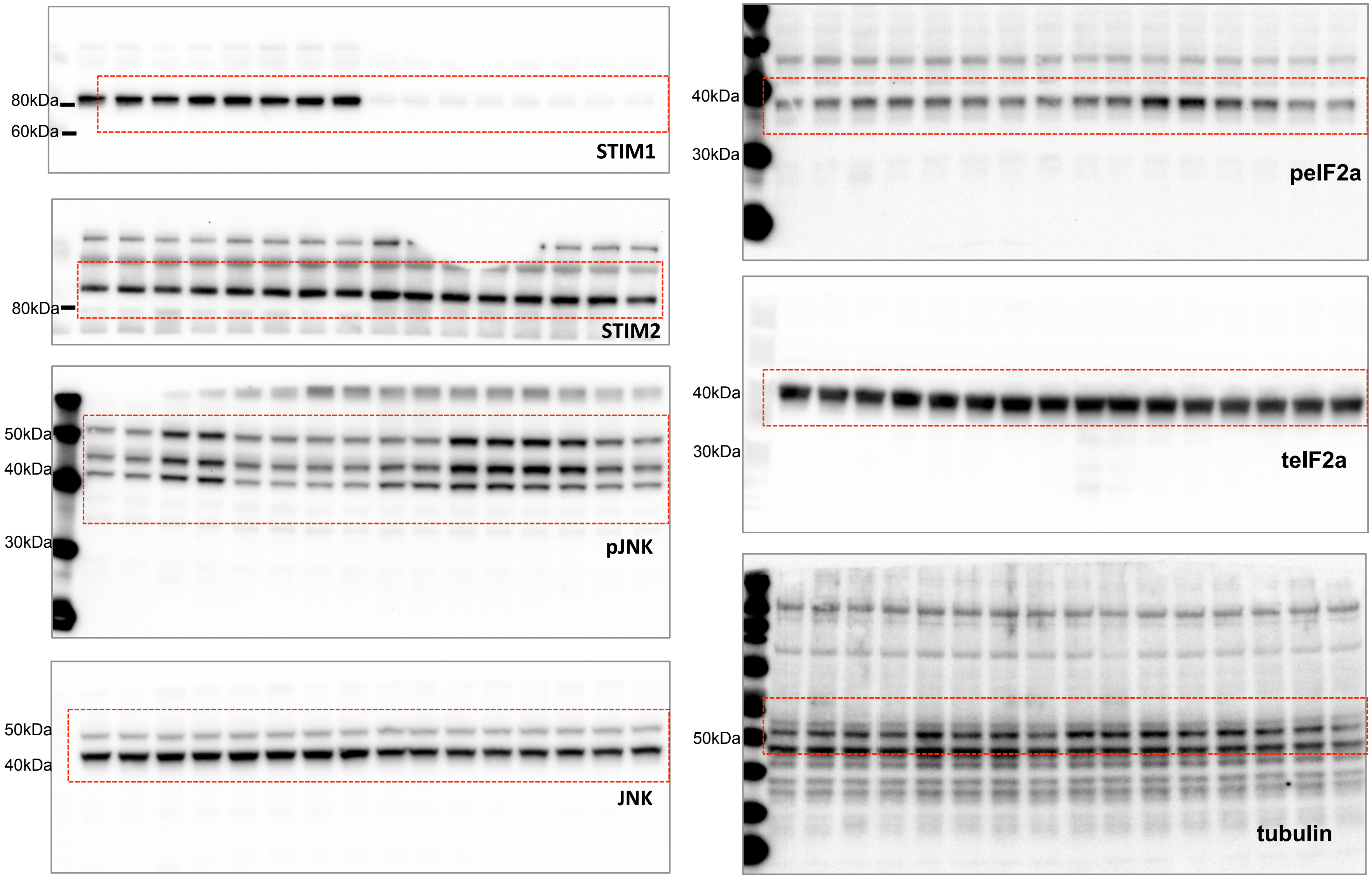
80kDa
60kDa
STIM1
80kDa
STIM2
50kDa
40kDa
30kDa
pJNK
50kDa
40kDa
JNK
40kDa
30kDa
peIF2a
40kDa
30kDa
teIF2a
50kDa
tubulin

Figure 3C

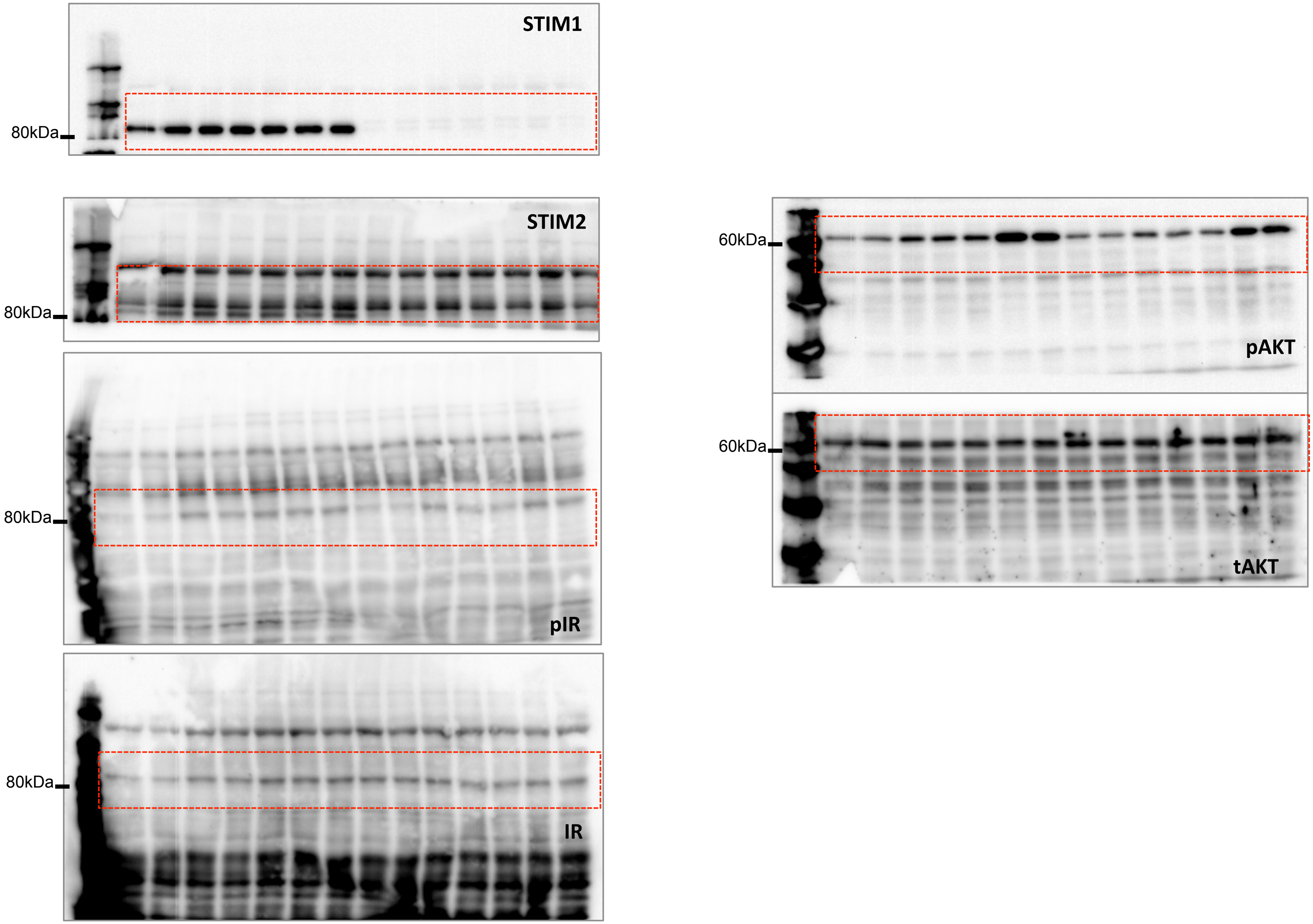
STIM1
80kDa
STIM2
80kDa
80kDa
pIR
80kDa
IR
60kDa
pAKT
60kDa
tAKT

Figure 3G

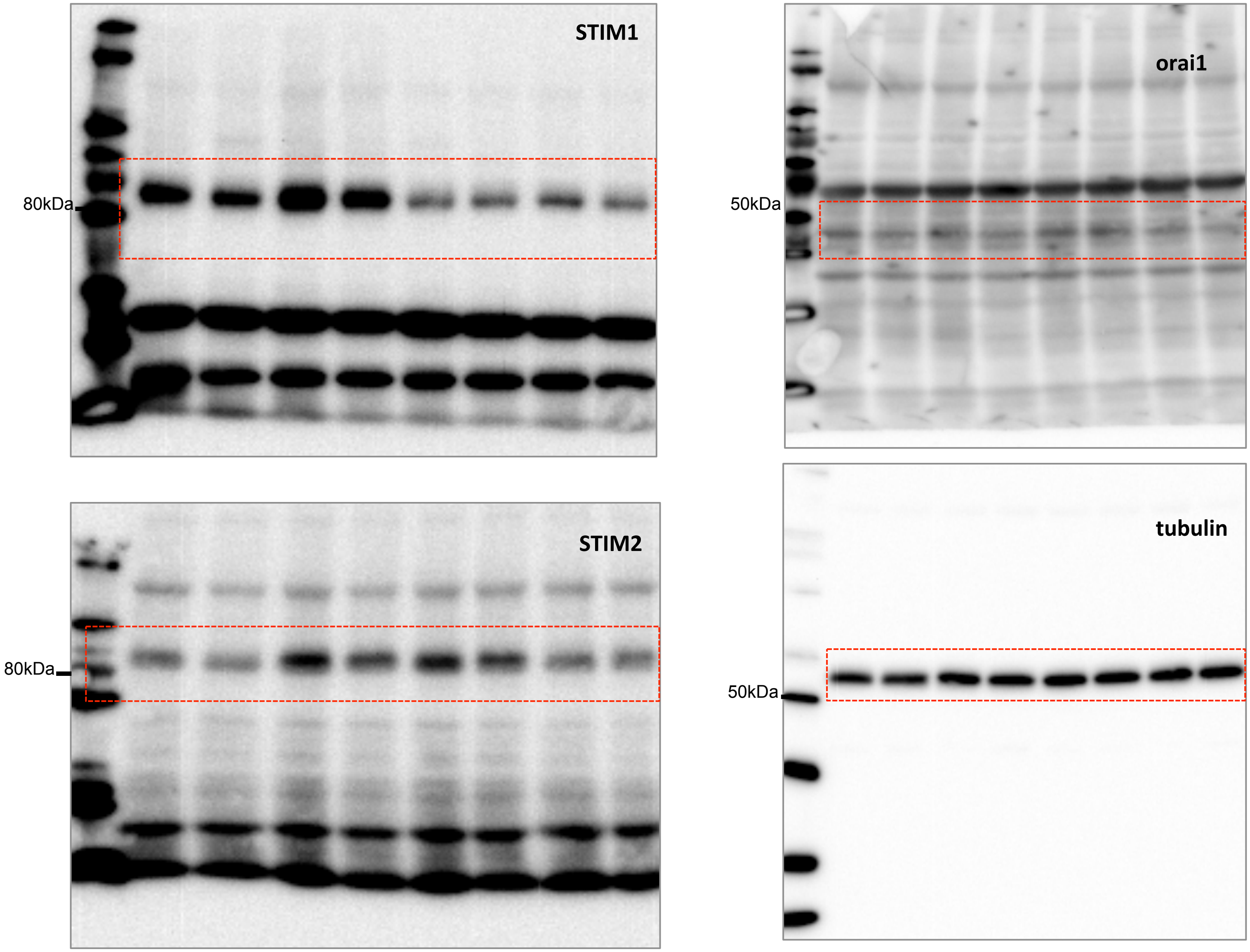

Figure 3 S1A

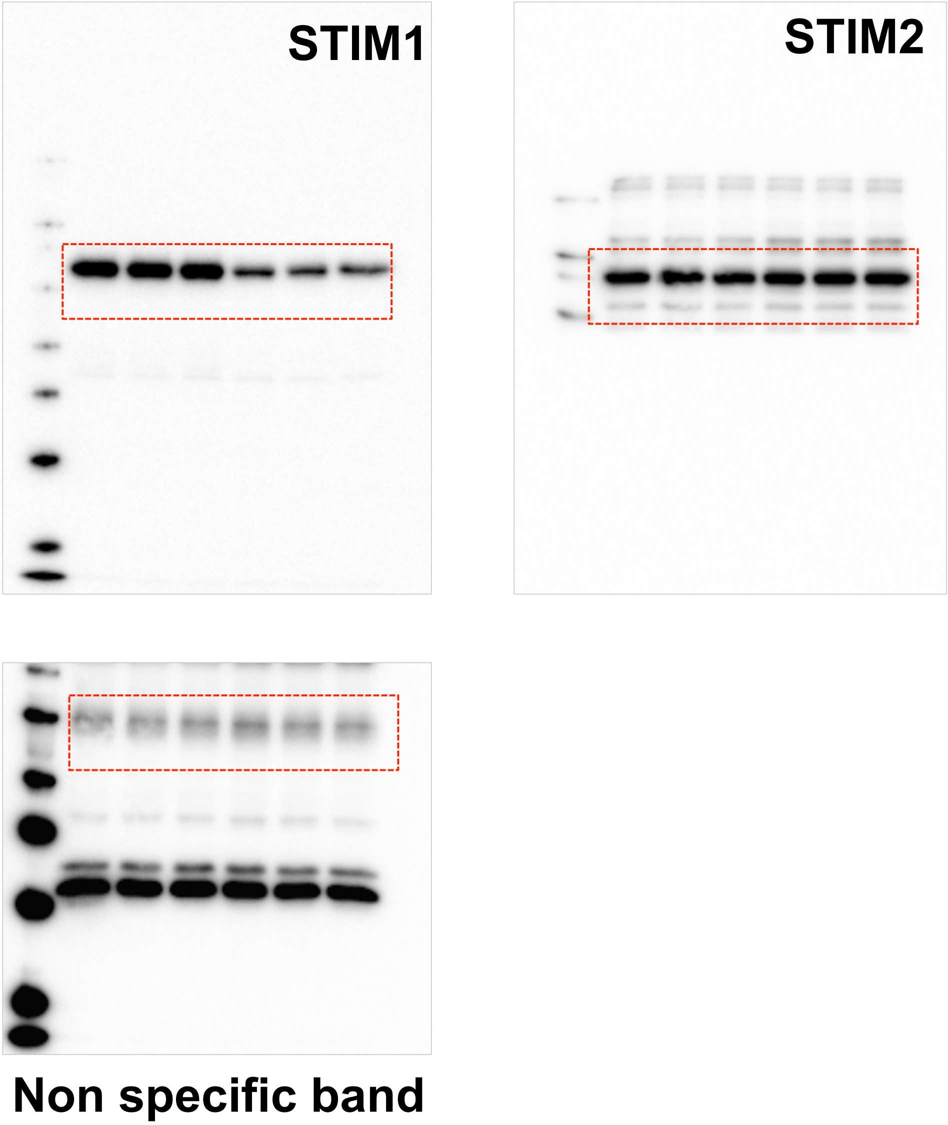

Figure 3 S1B

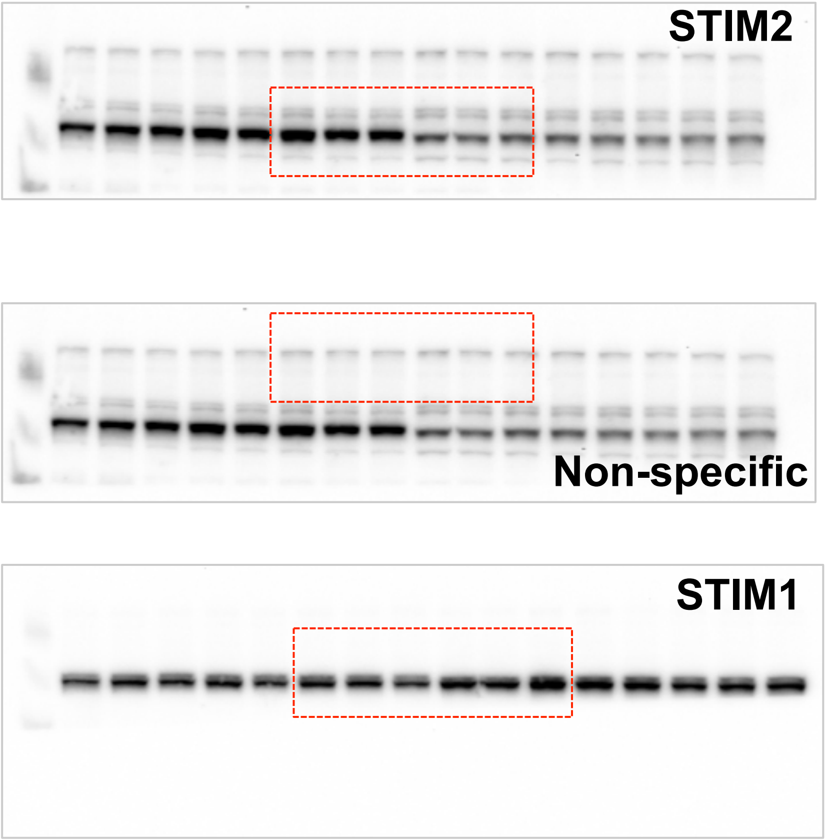

Figure 3 S1D

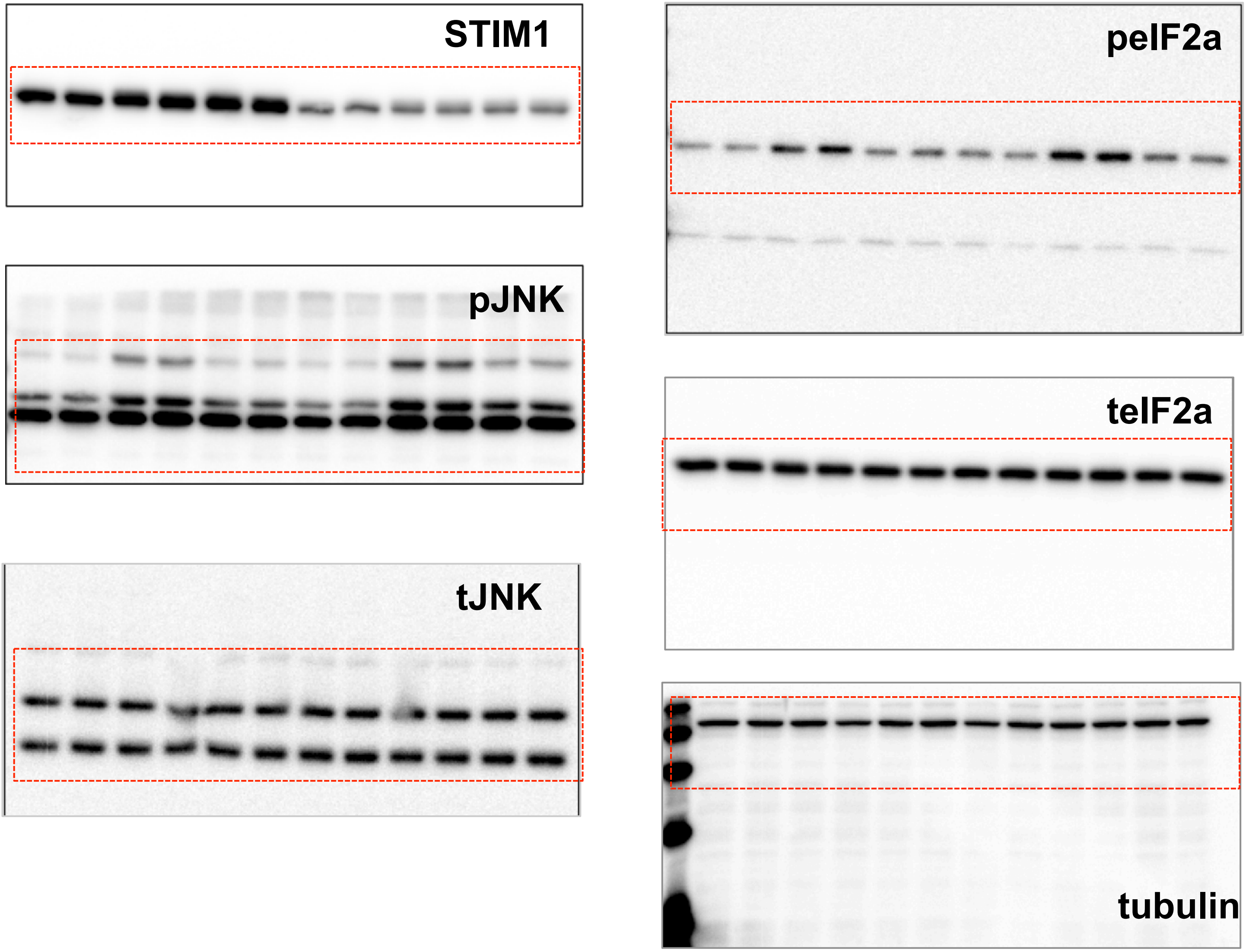

Figure 3 S1E

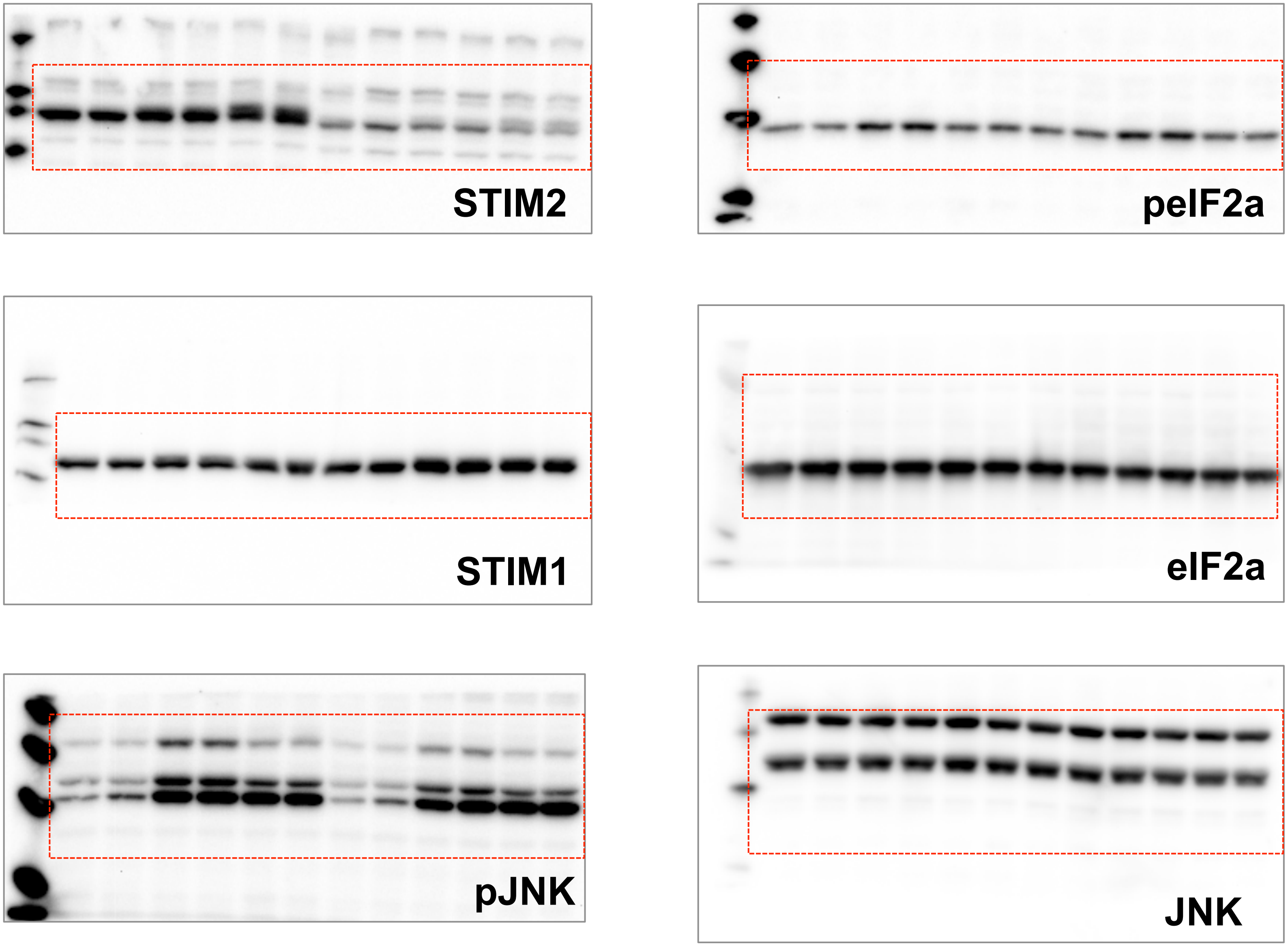

Figure 3 S1F

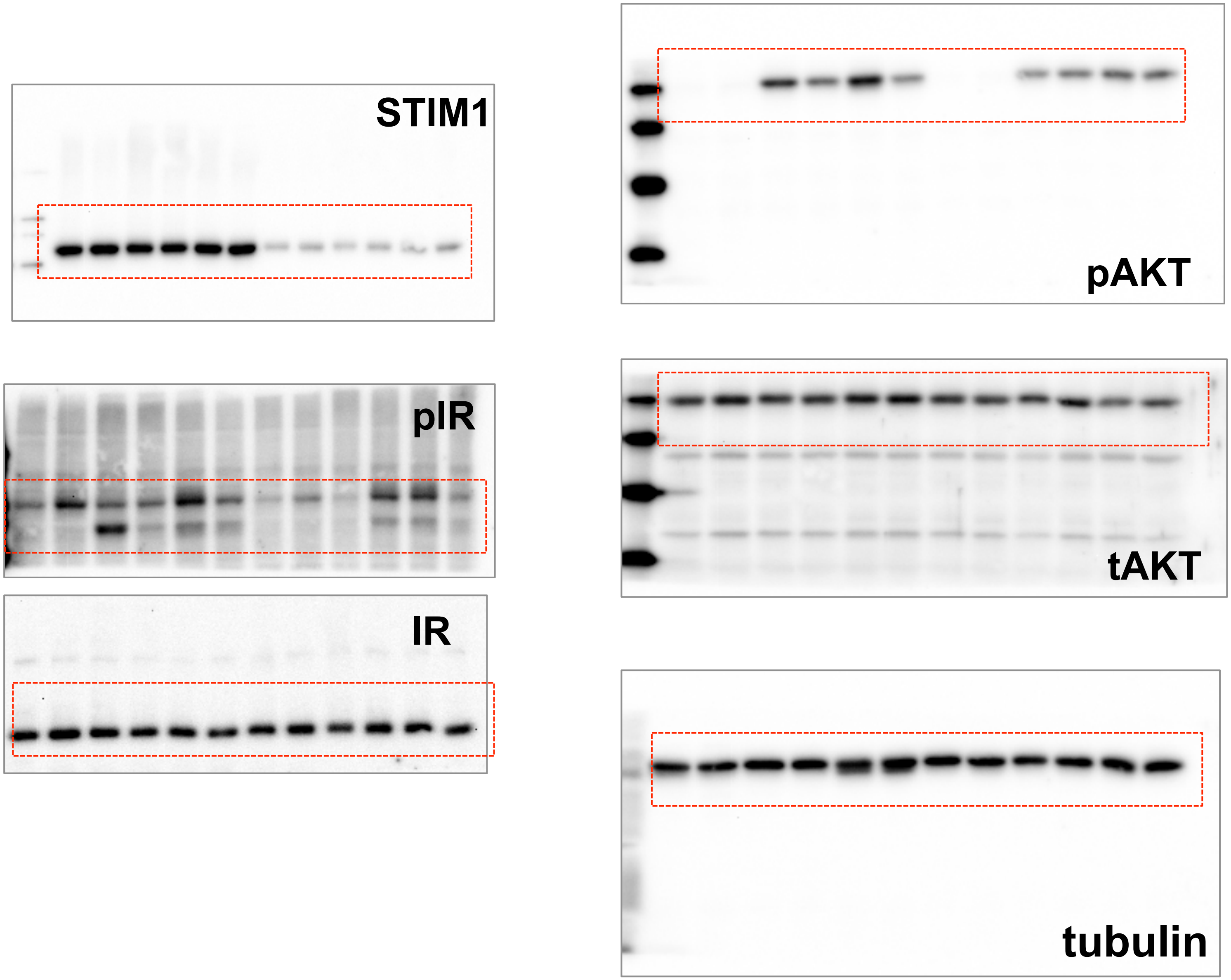

Figure 3 S1G

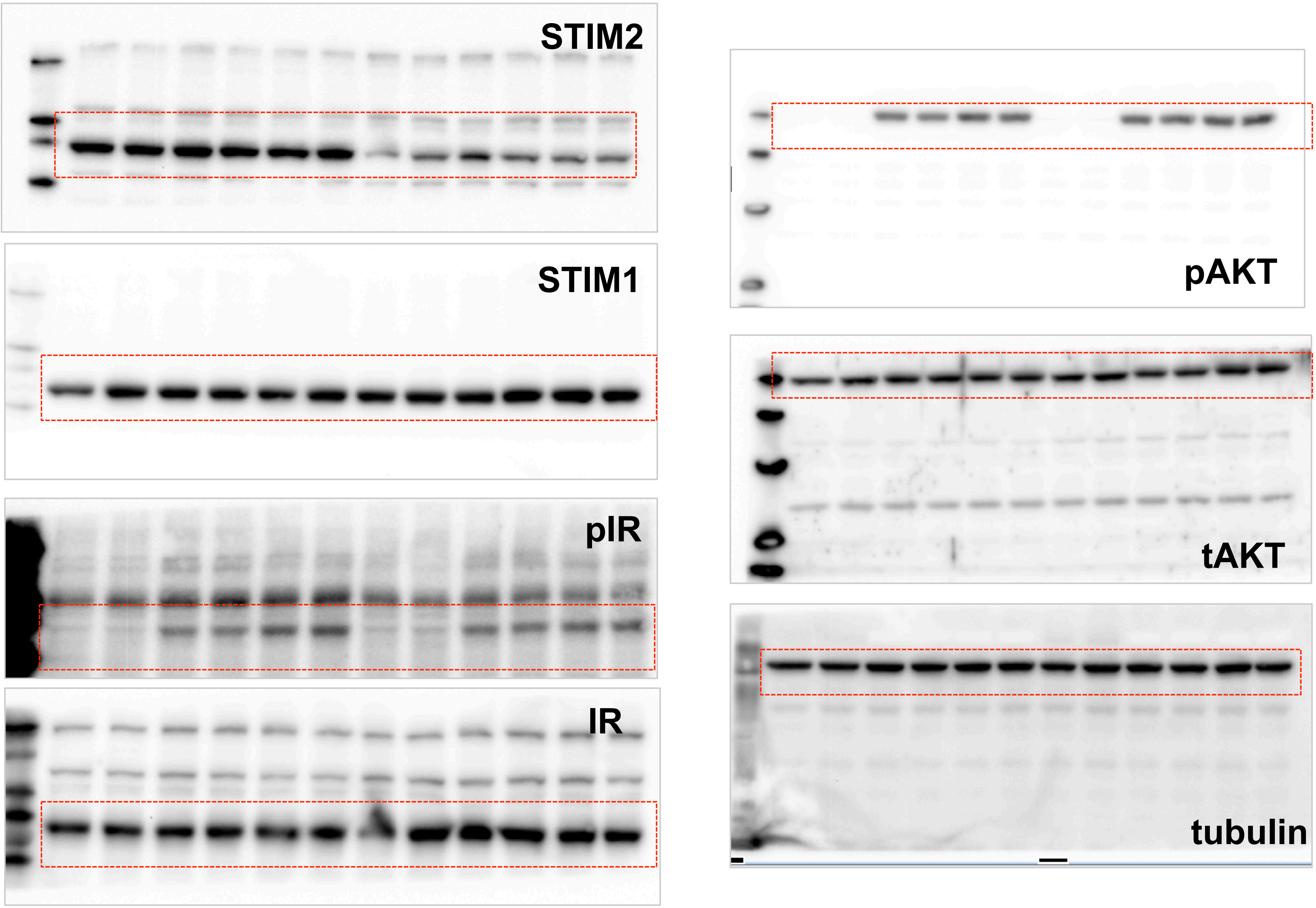

Figure 3 S2A

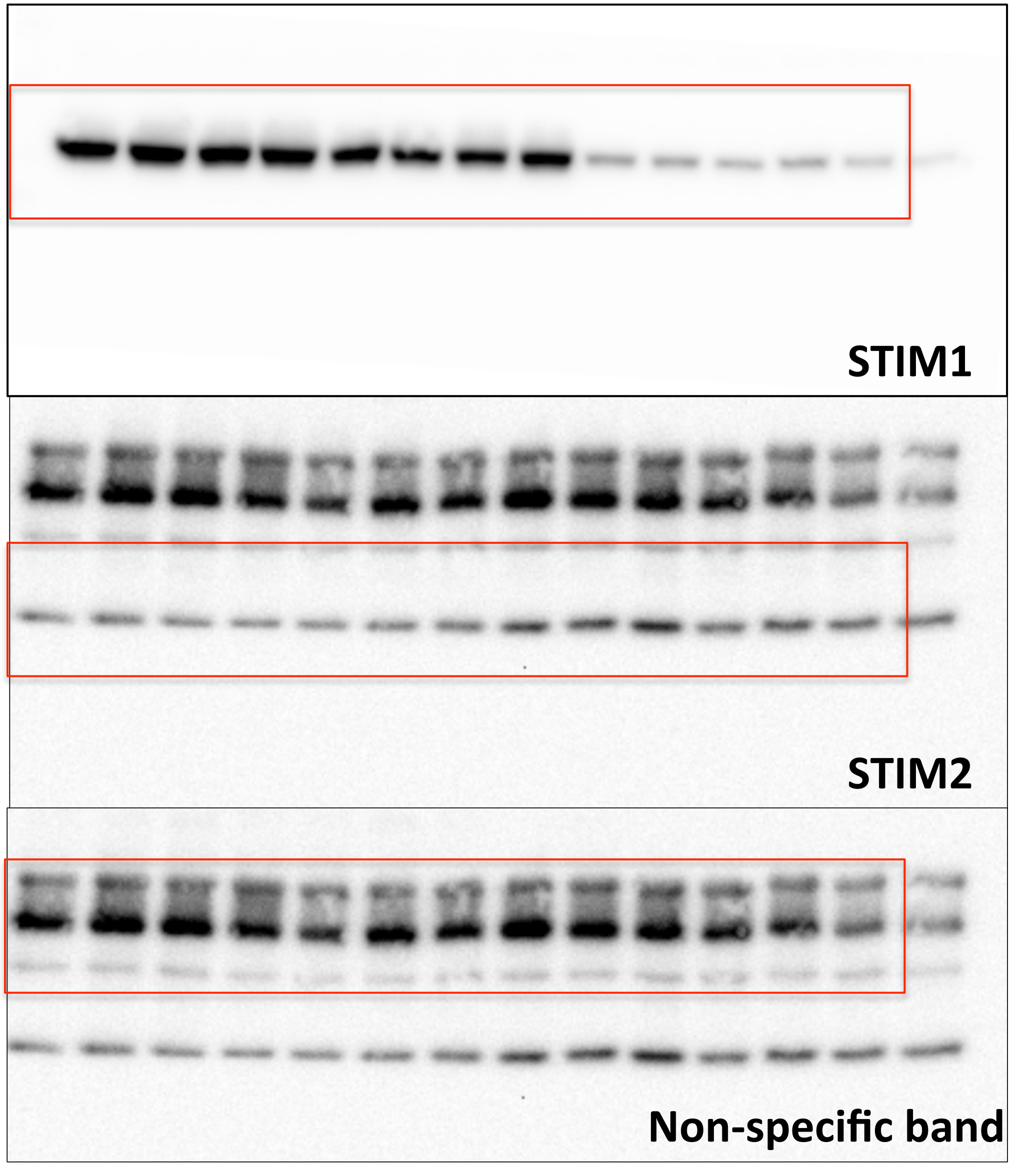

Figure 3 S2D

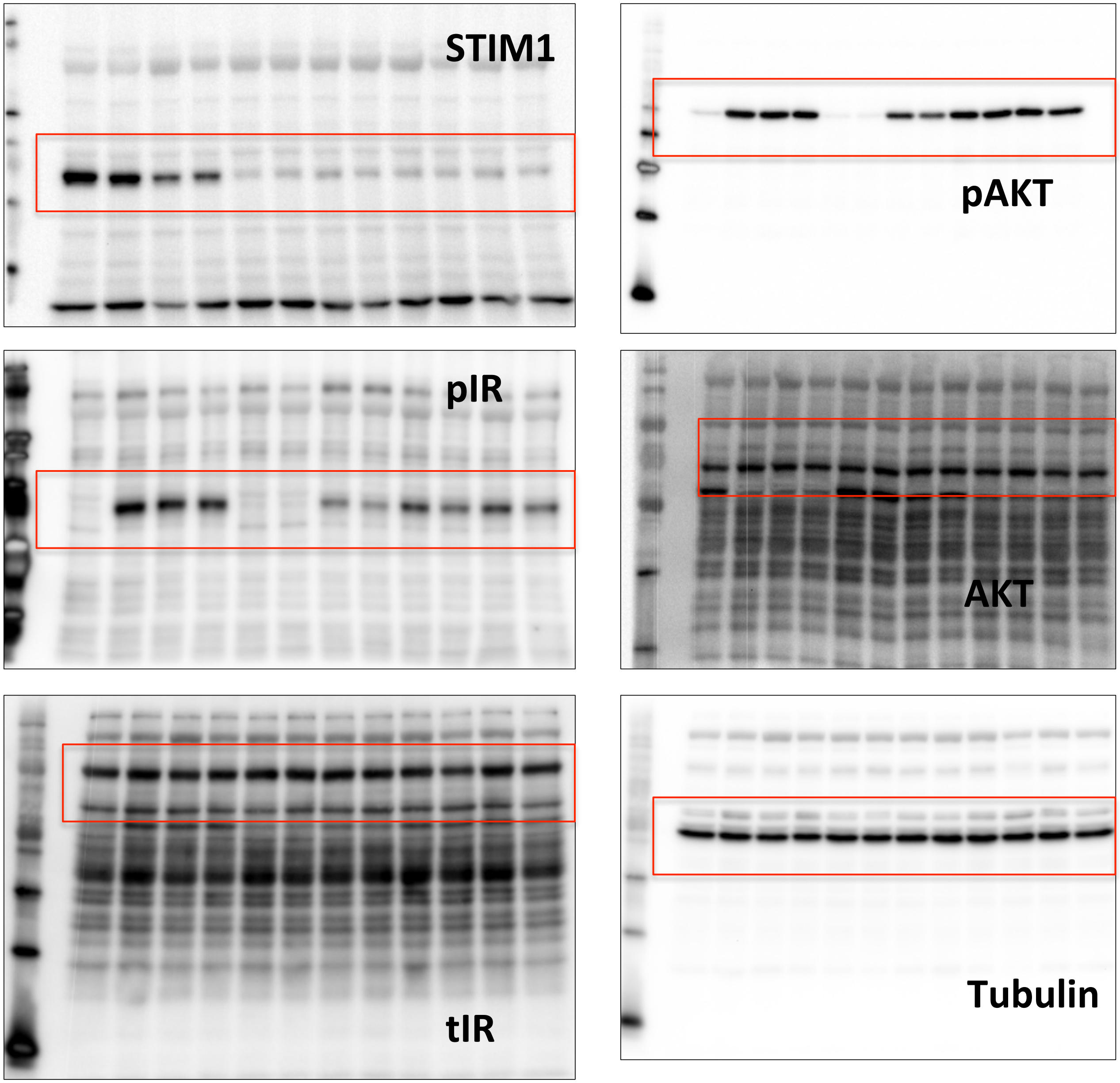

Figure 3 S2G

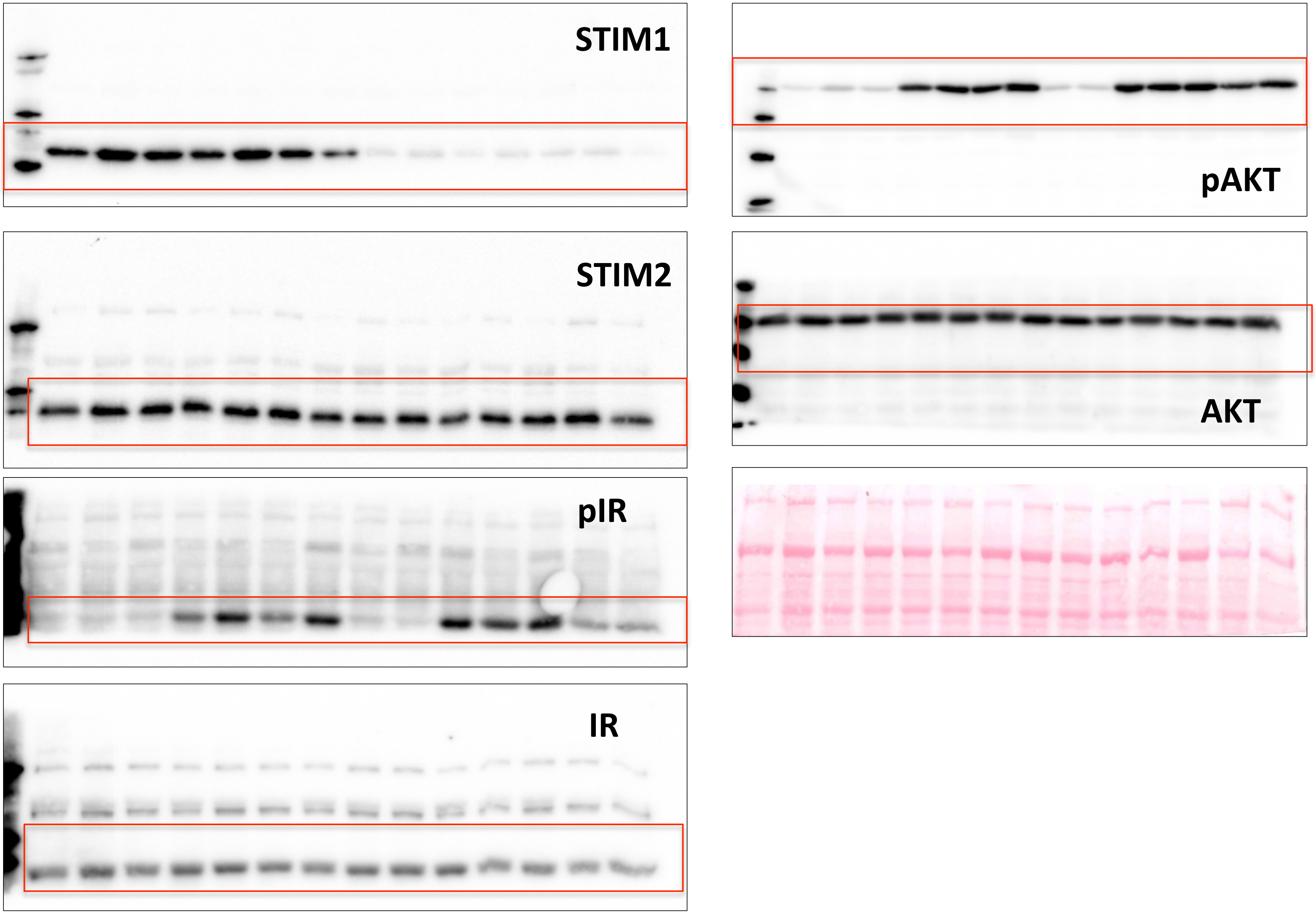

Figure 4A

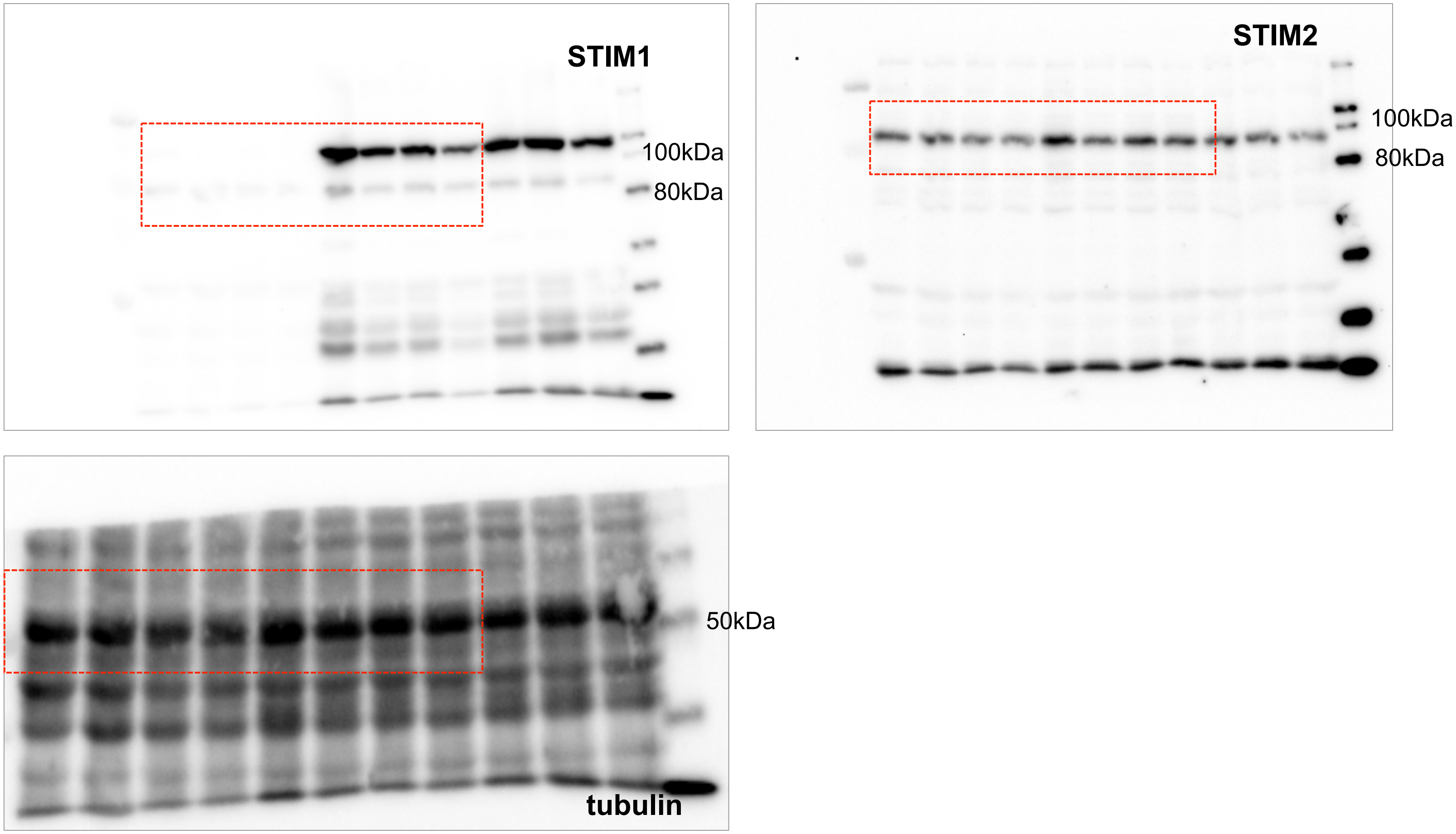

Figure 4F

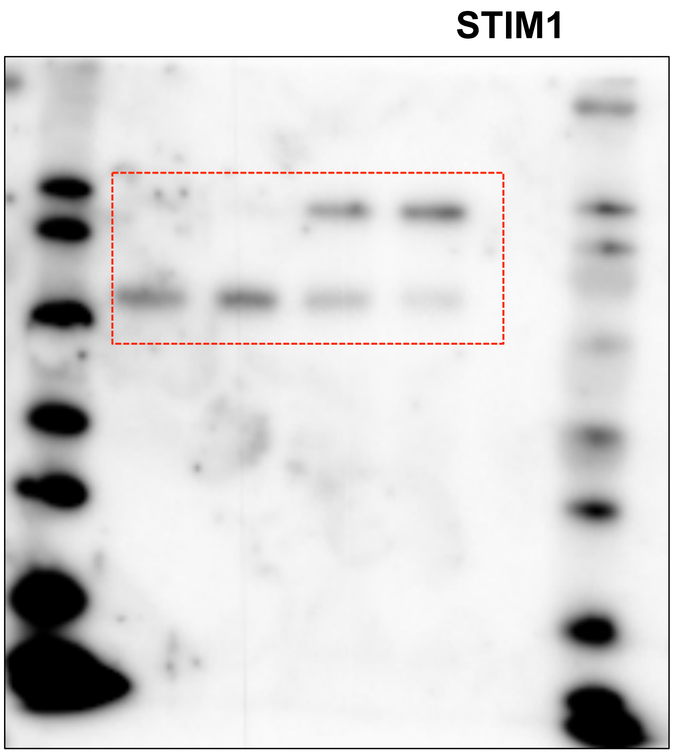

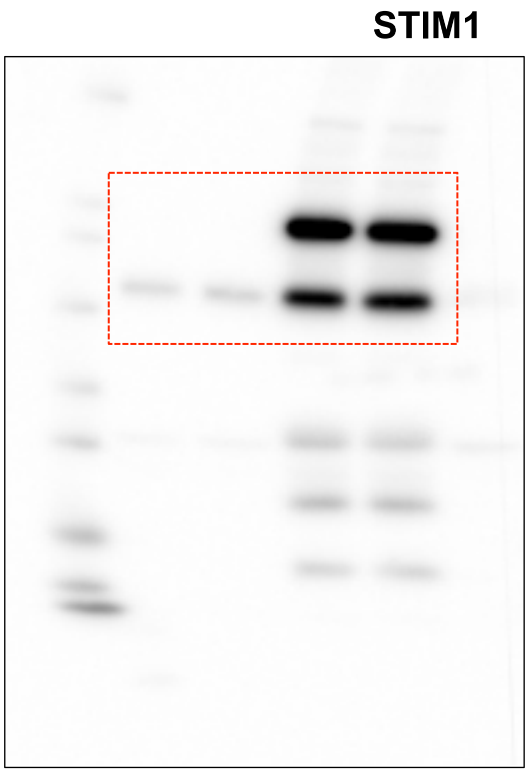

Figure 5C

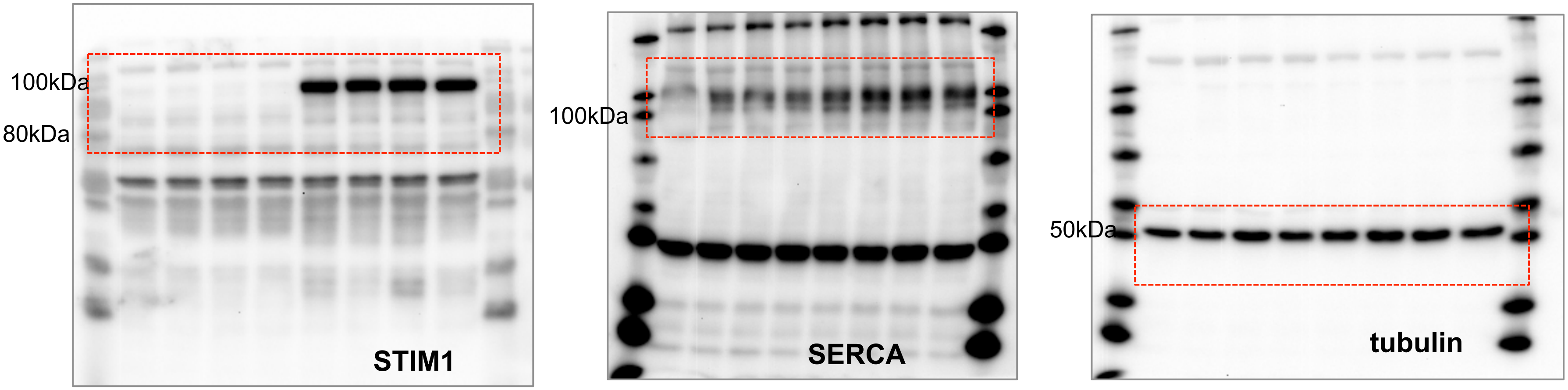

Figure 5E

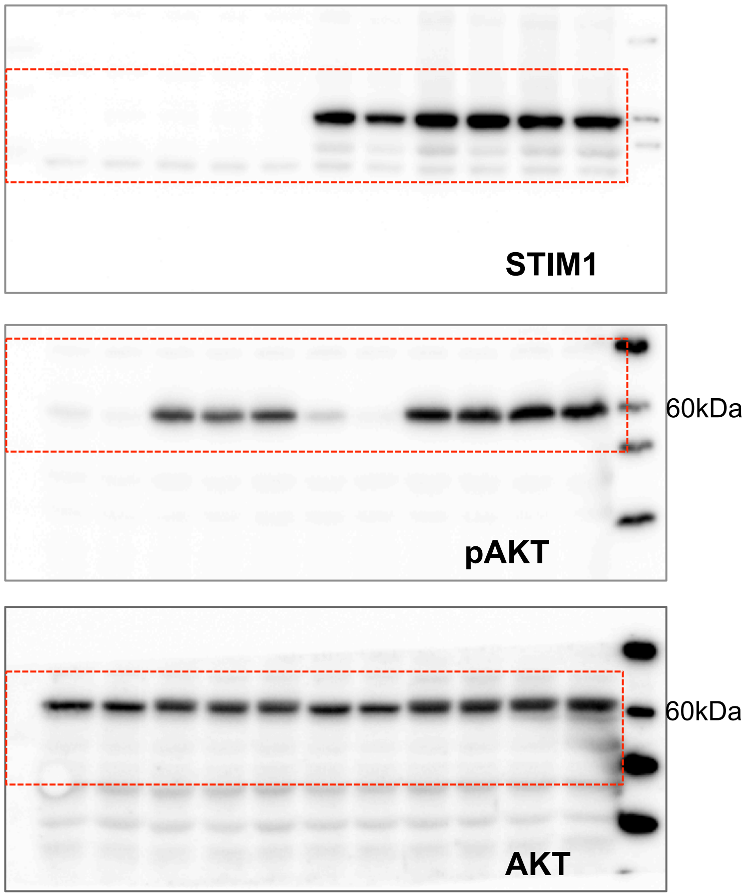

Supplement: Supplementary file 1. [file elife-29968-supp1.pdf]
